# Nutrigenomics in the context of evolution

**DOI:** 10.1016/j.redox.2023.102656

**Published:** 2023-03-11

**Authors:** Carsten Carlberg

**Affiliations:** a Institute of Animal Reproduction and Food Research, Polish Academy of Sciences, ul. Juliana Tuwima 10, PL-10748, Olsztyn, Poland; b School of Medicine, Institute of Biomedicine, University of Eastern Finland, FI-70211, Kuopio, Finland

**Keywords:** Human genome, Evolution, Lactase persistence, Disease risk, Nutrigenomics, Immunity

## Abstract

Nutrigenomics describes the interaction between nutrients and our genome. Since the origin of our species most of these nutrient-gene communication pathways have not changed. However, our genome experienced over the past 50,000 years a number of evolutionary pressures, which are based on the migration to new environments concerning geography and climate, the transition from hunter-gatherers to farmers including the zoonotic transfer of many pathogenic microbes and the rather recent change of societies to a preferentially sedentary lifestyle and the dominance of Western diet. Human populations responded to these challenges not only by specific anthropometric adaptations, such as skin color and body stature, but also through diversity in dietary intake and different resistance to complex diseases like the metabolic syndrome, cancer and immune disorders. The genetic basis of this adaptation process has been investigated by whole genome genotyping and sequencing including that of DNA extracted from ancient bones. In addition to genomic changes, also the programming of epigenomes in pre- and postnatal phases of life has an important contribution to the response to environmental changes. Thus, insight into the variation of our (epi)genome in the context of our individual's risk for developing complex diseases, helps to understand the evolutionary basis how and why we become ill. This review will discuss the relation of diet, modern environment and our (epi)genome including aspects of redox biology. This has numerous implications for the interpretation of the risks for disease and their prevention.

## Abbreviations

ADAMTS: ADAM metallopeptidase with thrombospondin type 1 motif
ADH: alcohol dehydrogenase
AMY: amylase
APEH: N-acylaminoacyl-peptide hydrolase
CCR5: C–C chemokine receptor 5
CNV: copy number variant
CVD: cardiovascular disease
CYP2E1: cytochrome P450 family 2 subfamily E member 1
DNMT: DNA methyltransferase
GWAS: genome-wide association study
HAT: histone acetyltransferase
HDAC: histone deacetylase
HIV-1: human immunodeficiency virus 1
Indel: insertion-deletion
KDM: lysine demethylase
KMT: lysine methyltransferase
LCT: lactase
LEPR: leptin receptor
MAF: minor allele variant
MAN2A1: mannosidase α, class 2A, member 1
MCM6: minichromosome maintenance complex component 6
NAD: nicotinamide adenine dinucleotide
NCOA1: nuclear receptor coactivator 1
OCA2: OCA2 melanosomal transmembrane protein
OR: odds ratio
PLAU: plasminogen activator, urokinase
Pol II: RNA polymerase II
POU2F1: POU class 2 homeobox 1
PPARD: peroxisome proliferator-activated receptor delta
ROS: reactive oxygen species
SARS-CoV-2: severe acute respiratory syndrome coronavirus 2
SI: sucrase-isomaltase
SLC: solute carrier family
SNP: single nucleotide polymorphism
SNV: single nucleotide variant
T2D: type 2 diabetes
T2T: telomer-to-telomer
TET: ten-eleven translocation
TSS: transcription start site
UBR1: ubiquitin protein ligase E3 component n-recognin 1

## 1 Introduction

Nutrition is an essential component of life, since it is composed of molecules that satisfy our body with its requirements of macro- and micronutrients [1]. In addition, these molecules affect our health, since some of them directly communicate with our genome and epigenome by regulating the activity of transcription factors and chromatin modifiers [2]. The complex relationship between nutrition and our (epi)genome is the core of nutrigenomics [3,4]. The daily diet-(epi)genome communication modulates the expression of genes in metabolic organs, such as in adipose tissue, skeletal muscle, liver and pancreas, as well as in the brain and the immune system. The cellular and molecular biology behind these gene regulatory processes maintain homeostasis of our body that prevent the onset of non-communicable diseases, such as obesity, type 2 diabetes (T2D), cardiovascular diseases (CVDs) and cancer.

In a given human population most anthropomorphic properties like height or eye color [5] as well as many physiological characteristics, such as lactase persistence [6] and the risk for developing diseases like T2D [7], exist in many forms. Since these traits are based on the expression and function of genes, the diversity is related to interindividual genomic and epigenomic variations. Accordingly, the members of a population display different levels of biological fitness like mating success, viability and fertility. Traits associated with increased fitness represent adaptations to the environment. This is often caused by evolutionary pressures, such as reduced availability of resources like food or threats like pathogens, and represents the basis of the evolutionary principle of “survival of the fittest”, as first formulated by Darwin [8].

In contrast, modern societies are characterized by intensive medical and social care for the individual. Taking care of the of less well-adapted members of a society is a hallmark of humanity and a significant advance of our species. Therefore, today's consequences of the principles of Darwinism in most cases do not apply anymore to us. However, infections with HIV-1 (human immunodeficiency virus 1) or the pandemic of SARS-CoV-2 (severe acute respiratory syndrome coronavirus 2) may represent exceptions. In any case, each of us is carrying in his/her individual version of the genome a history of the evolutionary past, which largely determines our personal susceptibility for various common diseases. This implies that the evolution of our species needs to be taken into consideration, in order to obtain the full potential of personalized medicine/nutrition [9].

Evolution is often based on positive natural selection, *i.e.*, on a process where an advantageous trait increases its prevalence in a population [10]. For example, individuals with a genetic advantage, such as a single nucleotide variant (SNV) that allows to express the *LCT* (lactase) gene in adult age can digest lactose and have with milk an important additional source of nutrition [11] (see section below). In the past, this implied that these individuals were more successful in reproduction, since they had a higher number of surviving children, which then also carry the same advantageous SNV. When the selection pressure persists, this increases over a number of generations the prevalence of the respective property until it becomes a major trait. Another prominent example of positive selection is the gene encoding for the CCR5 (C–C chemokine receptor 5) receptor, which is specifically expressed on T cells and essential for the entry of HIV-1. A rather common structural variant of the *CCR5* gene is a 32 base pair (bp) deletion, which significantly decreases the functionality of the protein and protects its carriers from HIV-1 infection [12,13]. However, the variant originated from Northern Europe more than 1000 years ago, *i.e.*, far earlier than HIV-1 circulated in humans. Therefore, more likely other infectious diseases, such as smallpox and/or the plague, created a selective pressure for the *CCR5* gene variant. In total, some 2000 human genes, *i.e.*, 10% of all protein-coding genes, may have undergone positive natural selection during the past 50,000 years [14]. This affected in particular genes related to the skin, the digestive tract and the immune system, since these organs are in a more intensive contact with the environment than others [15].

In addition to the process of positive selection, there are also randomly occurring mutations and recombination events of the genome, which can cause genetic drifts [16,17]. In humans, this so-called neutral evolution leads to a change of approximately 50 bp per individual and generation [18]. Although the respective genetic drifts are not under evolutionary pressure, they can reach high frequency in a population. Interestingly, in case of small populations or a weak selection pressure even deleterious alleles are able to reach high frequencies [19].

The statement “Nothing in biology makes sense except in the light of evolution” [20] implies that evolution, at least in the past, was also for humans a dominant driver in the development of any kind of biological process and its adaptation to environmental changes. Therefore, the evolutionary history of our species needs to be considered also in the field of nutrigenomics. Accordingly, the focus of this review is to provide an overview on the relation of nutrigenomics and evolution.

## 2 The human genome and its variation

Thousands of complex phenotypic traits determine our physical appearance and what is our risk to develop non-communicable diseases. In addition, each of these traits relates to dozens to hundreds of variants in the genome as well as to environmental influences affecting our epigenomes [21] (see section below). In general, the genetic architecture of a trait depends on [22]:

• the number of variants that influence the respective phenotype
• the relative magnitude of the effects of the different variants on the trait
• the frequency of the traits in the population
• the interference of the traits with each other and the environment.

Importantly, most of the variants, which are associated with traits, are not found at protein-coding regions but rather affect regulatory genomic regions, *e.g.*, transcription factor binding sites [23]. The latter sites are often referred to as regulatory SNVs, master examples of which will be discussed in the context of lactase persistence.

The Human Genome Project (www.genome.gov/10001772) resulted in 2001 in the release of the first reference haploid sequence of our genome [24,25]. However, it took more than 20 years until all gaps in the sequence could be filled and the telomer-to-telomer (T2T) final version of our genome was published [26]. It comprises 2 × 3.05 billion bp (Gb) on 46 chromosomes (2 × 22 autosomes and XY for males or XX for females). The haploid genome contains 63,494 genes, of which only 19,969 are protein-coding, *i.e.*, the majority of human genes encode for non-coding RNAs with structural and regulatory functions. The human reference genome had been assembled based on individuals from European ancestry, but there are initiatives to include genomes from people with African and Asian ancestry [27,28], in order to have a better representation of all major populations.

Each of us is carrying a number of variants in comparison to the reference genome. They represent in total some 1% of the whole sequence. These are 4–5 million SNVs, where exactly one nucleotide is altered, and some 600,000 structural variants that often affect more than one nucleotide [29]. Most of the latter are insertion-deletions (Indels), where 1–49 bp are added or removed, respectively. In addition, per individual there are some 1000 genomic regions carrying copy number variations (CNVs), where DNA stretches from 50 bp up to 15 million bp (Mb) in length are either inserted or deleted.

Variants to the human genome, which have a minor allele frequency (MAF) of at least 1%, are called common, when their MAF is below 1% [30] (www.ncbi.nlm.nih.gov/SNP). Approximately 7 million human SNVs are single nucleotide polymorphisms (SNPs), because they have a MAF higher than 5%. The 1000 Genomes Project (www.internationalgenome.org) and other larger whole genome sequencing projects, such as the TOPMed program [31] and Genomics England [32], have provided more than 100,000 whole genome sequences, on the basis of which nearly 500 million SNVs were identified [33]. Concerning SNVs the average difference between two unrelated individuals is 0.1%, which in comparison to other species is a very low value and related to the recent origin of non-African *homo sapiens* populations from a small founding group [34,35] (see section below).

Within the coding sequence the effect of a SNV can either be synonymous, when it does not alter the encoded protein, or non-synonymous, when it causes a change in a single amino acid (missense) or introduces a premature stop codon (nonsense). Each individual's genome contains some 150 SNVs that cause protein truncation and 10,000 SNVs leading to changes in amino acids, while 500,000 SNVs modulate the binding capacity of transcription factor binding sites [33]. Within exons Indels and CNVs can cause frameshift mutations, *i.e.*, a complete change in the protein sequence, and within introns CNVs may affect the splicing process. Importantly, each of us carries 50–100 heterozygous variants in our genome that in a homozygous setting can cause monogenetic disorders.

In the last 20 years the impact of variations of the human genome on the risk for diseases has been primarily investigated by genome-wide association studies (GWASs) using arrays of thousands to millions of SNPs [36]. This method is now replaced by whole exome or whole genome sequencing [37]. The studies aim for a statistically significant association between a genetic variant and the occurrence of a disease. The average SNV density of 1 in 1000 nucleotides suggests that these studies require testing of millions of genetic variants per individual as well as samples from thousands of individuals. GWASs with several thousand individuals could identify odds ratios (ORs) of 1.5, *i.e.*, a 50% increased risk for the tested disease. Larger sample sizes are often achieved by pooling several GWASs through meta-analyses. For example, with more than 50,000 individuals one can determine increased risks by 10%, *i.e.*, ORs of 1.1. Disease- and trait-associated genomic loci can be found in the database GWAS Catalog (www.ebi.ac.uk/gwas).

**Fig. 1:**
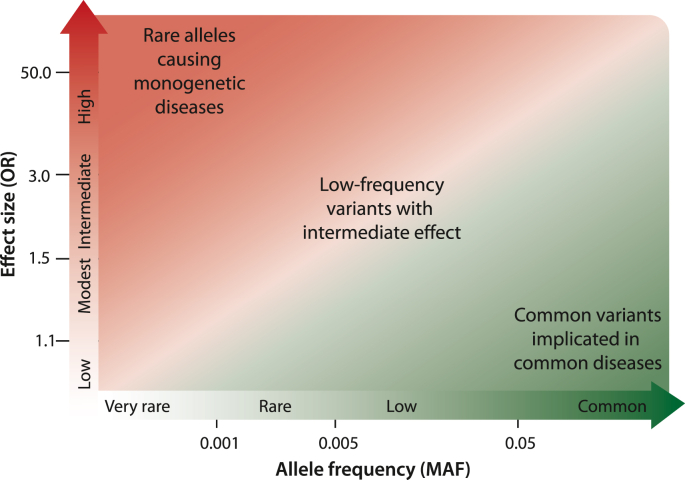
**Risk allele frequency of genetic variants.** ORs indicate the strength of a genetic effect. Main emphasis on the identification of associations within the diagonal box. Whole genome sequencing of large numbers of individuals identifies far more low frequency SNVs with intermediate ORs (**center**).

Mendelian disorders, such as cystic fibrosis or Huntington's disease, are monogenetic, *i.e.*, in these cases a single homozygous SNV with a strong effect can explain the occurrence of the rare disease [38] (Fig. 1, left). In contrast, common diseases like T2D, CVDs or cancer have a multigenic basis, *i.e.*, they are based on a multitude of SNVs, each of which having only a minor impact [39] (Fig. 1, right). For example, common traits like body height are determined by at least 180 genomic regions [40]. Interestingly, improved quality and quantity of nutrition had a major environmental influence, which in Europe led within the last few generations to 10 additional centimeters in average height [41].

In summary, the variations of our genome represent the genetic component of our risk for diseases as well as for anthropometric traits.

## 3 The impact of epigenetics

Despite the successes in revealing the association of numerous variants of the human genome with complex traits, only some 10% of the heritability of most complex diseases can be explained by genetics [42]. This suggests that based on SNV analysis one cannot reliably estimate the risk of an individual for a particular disease. The only well-known exceptions are age-related macular degeneration [43] and type 1 diabetes [44]. In contrast, the heritability of only 20% of coronary heart disease cases is explained by more than 60 genetic loci [45], 20% of T2D risk by some 300 loci [46] or 20% of breast cancer risk by some 170 loci [47]. It is possible that rare variants with high ORs may explain some of the missing heritability [48], but environmental exposures have the main impact. Many of latter are related to nutritional molecules or their metabolites that affect the epigenome [49].

The physical expression of the epigenome is chromatin, which is a complex of nucleosome-forming histone proteins and genomic DNA [50]. Epigenetics is defined as functionally relevant changes of chromatin that do not affect the sequence of the genome [51]. For example, during embryogenesis, when totipotent embryonic stem cells differentiate *via* various progenitor cells into all 400 tissues and cell types forming our body [52], there are cascades of changes in DNA and histone methylations, in the context of which the cells are epigenetically programmed. Comparable epigenetic programming takes place when adult stem cells in bone marrow, colon and skin, give rise to new blood cells, enterocytes and keratinocytes, respectively.

Epigenetic changes are mediated by chromatin modifying enzymes. For example, histone acetyltransferases (HATs) add acetyl groups to lysines of histone proteins, while histone deacetylases (HDACs) remove them. Comparably, lysine methyltransferases (KMTs) provide histones with methyl groups and lysines demethylases (KDMs) erase them. DNA can be methylated at cytosines through the action of DNA methyltransferases (DNMTs) and TET (ten-eleven translocation) enzymes, which initiate the removal of the methyl groups. The relative activity of these enzymes determines the level of accessibility of genomic regions, *i.e.*, whether transcription factors can bind to enhancer regions and transcription start sites (TSSs) are accessible to RNA polymerase II (Pol II). Interestingly, many chromatin modifying enzymes use intermediate metabolites of energy metabolism as cofactors, such NAD^+^ (nicotinamide adenine dinucleotide) for some HDACs, acetyl-CoA by HATs and α-ketoglutarate by KDMs, *i.e.* the activity of chromatin modifiers largely depends on the redox state [53] and the metabolic status of the cell [54]. Interestingly, the antioxidant vitamin C is a cofactor to dioxygenases, such as some members of the KDM family and TET enzymes [55]. This makes critical steps in epigenetic regulation, such as the demethylation of histones and genomic DNA, dependent on a compound that humans and many other mammals have to take up from diet, because they are incapable to synthesize it themselves.

Some patterns of DNA methylation or histone modifications last for days, months or even years [56]. In this way, the epigenome is able to preserve effects of cellular perturbations, such as the supply with nutrients, as epigenetic drifts, which represent a type of memory [57,58]. When somatic cells divide, these epigenetic drifts may be inherited to daughter cells and *via* germ cells the epigenetic memory can be transferred even to the next generation [59].

Taken together, many epigenetic variants are the result of responses to environmental changes, such as the supply with nutrients. In this way, the epigenome is able the react faster to evolutionary pressures than the genome and may explain major parts of the missing heritability.

## 4 Migration of *homo sapiens* and the diversity of human populations

At their origin in Africa, hominins lost some 2 million years ago most of their body hair [60], in order to improve *via* more efficient sweating their endurance performance [61]. Their initial pale skin developed an intensive pigmentation, in order to protect from sunburn and UV-induced cancer [62]. The pigmentation intensity of skin, eyes and hair can be explained by SNVs in genes encoding for key proteins of melanin synthesis in melanosomes [63]. For example, the pale skin of European populations is explained primarily by SNVs related to the genes *OCA2* (OCA2 melanosomal transmembrane protein), *SLC45A2* (solute carrier family 45 member A2) and *SLC24A5* [64,65]. The SNVs cause a loss of function in the encoded pH regulator, ion transporter and potassium-dependent sodium/calcium exchanger, respectively, and lead to a reduced production of the black/brown eumelanin in melanosomes [63,65]. A reduced skin pigmentation may increase the efficiency of UV-B-induced vitamin D_3_ production, which is important for populations living at higher latitudes [66]. Vitamin D is, in contrast to vitamins C and E, not a classical antioxidant, *i.e.*, it is not a scavenger for reactive oxygen species (ROS). However, UV-B absorption by the vitamin D_3_ precursor 7-dehydrocholesterol shields cholesterol-producing animals and plants against radiation damage. Therefore, even simple eukaryotes like phytoplankton produce vitamin D_3_ although they do not use it for endocrine function purposes [67].

**Fig. 2:**
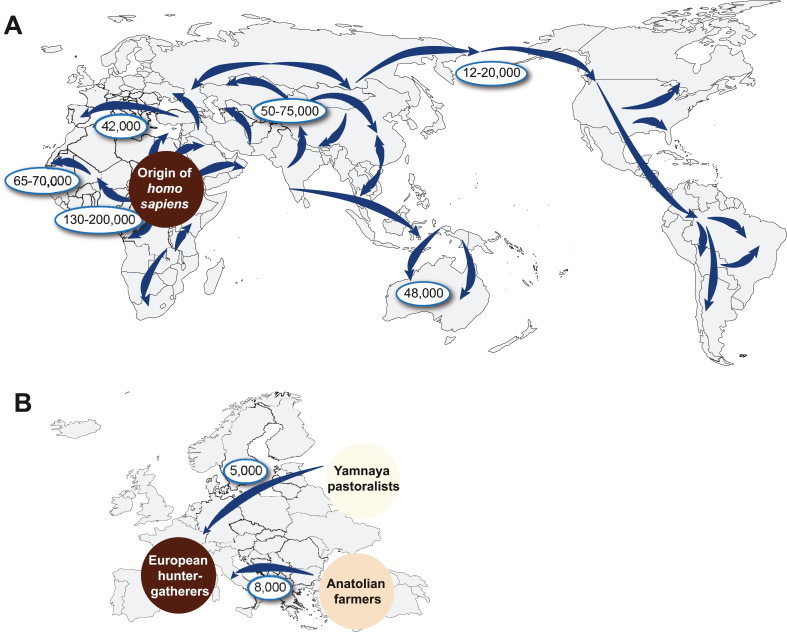
***Homo sapiens* migrations.** Anatomically modern humans developed in East Africa and spread first over the whole continent before they started some 75,000 years ago to migrate to Asia. From there they settled in Oceania, Europe and the Americas.

Anatomically modern humans (*Homo sapiens*) evolved in East Africa some 250,000 years ago [68,69] and spread first over the whole African continent (Fig. 2A). About 75,000 years ago a subgroup of them migrated first to Asia and then further to Oceania, Europe and the Americas [65,70]. The number of modern humans who left Africa at that time and got the ancestors of all *homo sapiens* at the other continents, was far smaller than those, who stayed in Africa. This created a bottleneck and reduced the genetic diversity of the non-African populations [35]. In addition, after migration a number of human populations became isolated due to geographic, language and political barriers, *i.e.*, over time human genetic variation diverted geographically.

The persistent arrival of *homo sapiens* in Europe was about 42,000 years ago [71,72]. Both in Europe and in Asia modern humans met ancestral Neanderthal and Denisovan hominins, which they outnumbered by interbreeding [[73], [74], [75]]. Due to this so-called introgression process, 1.5–2.1% of the genome of modern humans in Europe have Neanderthal origin [76]. In contrast, present Southeast Asians have in average only 0.1% of their genome from Denisovans, while the rate is 3.5% in some Oceanian populations [77]. Many of the genes that we inherited from Neanderthals affect physiological systems all over our body and raise the risk of a number of diseases. However, some Neanderthal gene variations also show beneficial effects, *e.g*., those boosting the immune system. In Europe, hunter-gatherers lived first in the ice-free southwest of the continent [78] and 11–12,000 years ago, after the end of the ice age, some of them followed the migration of animal herds to northern Europe [71]. Archeogenomic data from hundreds of individuals living between 8500 and 2300 years ago [79] suggest that some 8400 to 6000 years ago a wave of people from northwestern Anatolia arrived in southern Europe (Fig. 2B). These so-called Anatolian farmers introduced to the hunter-gatherers the concept of agriculture and started in this way the Neolithic revolution. The latter is characterized by giving up the nomadic lifestyle and the domestication of a number of plant and animal species. Some 5000 years ago, a second wave of migrants, the Yamnaya pastoralists, arrived in Europe. They originated from the Eurasian steppe, introduced the wheel, the horse and their Indo-European languages to the European populations and settled preferentially in northern Europe. Both groups of migrants had lighter skin than the hunter-gatherers [[80], [81], [82]], *i.e.*, the light skin color of today's Europeans became frequent only within the past 5000 years [65,83,84]. This phenotypic adaptation is primarily based on non-synonymous SNVs of the genes *SLC24A5* and *SLC45A2* [65].

The individual admixtures of the genomic variants originating from hunter-gatherers, Yamnaya pastoralists and Anatolian farmers explain the variation in skin color as well as many other traits of the European population [81,[85], [86], [87]]. This includes also the individual's genetic risk for common diseases [88,89]. The neolithic revolution caused a rapid increase in population size in Europe [90,91], such as the use of milk products as food source for adults and the rise of agriculture [92] (see section below). Some 500 years ago major voluntary and involuntary (slaves) migration started, in particular between Europe, Africa and the Americas. This led to significant population admixtures, in particular in the Americas, but also in other parts of the world.

In summary, after their worldwide expansion the population size of modern humans was growing exponentially [93]. This growth modified the genetic architecture of traits and generated many low-frequency variants of the human genome [94]. Moreover, this caused substantial differences in allele frequency between populations, some of which are relevant to disease risk [95].

## 5 Evolution of human nutrition

Until some 10,000 years ago all members of our species were hunters and gatherers, *i.e.*, they were eating wild animals and plants with an estimated 20%/80% ratio [96] (Table 1). This primarily plant-based diet had rather low energy density, had medium fiber content and was low in fat. Moreover, the paleolithic diet did not contain any sugar and was low in salt but had a high micronutrient density and antioxidant capacity. Since *homo sapiens* had some 240,000 years, *i.e.*, about 10,000 generations, time to adapt to this type of food, it can be considered as the reference, to which the biochemistry of our body has accommodated.

**Table 1:** **Evolution of human nutrition.** Human diet changed in the shift from hunter and gatherers to farmers. The next change was introduced by the industrial revolution, but the most drastic change in diet was in modern times.

Time period	Diet	Nutritional characteristics
**Paleolithic era** (more than 10,000 years ago)	Wild animals and plants	Low energy density, medium fiber content, no sugar, low glycemic load, low fat and low salt
**Agricultural revolution** (starting 10,000 years ago)	Domesticated animals and plants. Use of fermented foods and beverages	Medium energy density, high fiber content, low sugar, medium glycemic load, medium fat and high salt
**Industrial revolution** (starting 250 years ago)	Reliance of refined grains and oils, fatty meat, alcoholic beverages	High energy density, medium fiber content, medium sugar, high glycemic load, high fat, and high salt
**Modern era** (starting 50 years ago)	Industrially produced foods, fatty meat, alcoholic beverages. Consumption of fast food	Very high energy density, low fiber content, high sugar, very high fat, very high glycemic load and high salt

Giving up the hunter and gatherer habit and becoming farmers started in different regions of the world as early as 10,000 years ago. This neolithic revolution is characterized by the use of domesticated plants and animals [97]. It shifted the dietary pattern by introducing sugar and using salt for conservation. The more energy-rich diet led to a significant increase in population density. However, the side effect of living in close contact with many other individuals as well as with a number of domesticated animal species was an increased burden of infectious diseases, many of which derive from zoonotic transition [98] (see section below). Thus, the change in diet as well as infectious diseases created evolutionary pressures that pushed the rather rapid adaption of a few key genes [99] (see section below).

The industrial revolution, which started some 250 years ago, resulted in a large number of machines and transport vehicles, so that individuals had to invest less and less physical activity into daily work. In parallel, refined foods, such as oils and grains, were used, so that the fiber content of diet decreased and the sugar and fat load increased. Since some 50 years, a dietary pattern that is characterized by high intakes of pre-packaged foods, refined grains, red meat, processed meat, high-sugar drinks, sweets, fried foods, butter and other high-fat dairy products is referred to as Western diet [100]. It was distributed by US American fast-food and supermarket chains to Europe and has arrived in nearly all countries and human populations. Special impact had high-fructose corn syrup, which is used as a replacement of sucrose as a sweetener [101]. Thus, in modern times the average fiber content of our diet further decreased, while the sugar and fat load increased. Today's diet has a glycemic load higher than ever in our history, while in parallel due to technical revolutions in transportation and computerization physical activity further decreased. Therefore, an increasing proportion of the worldwide population receives a positive energy balance, which is the main reason for the worldwide epidemic of overweight and obesity [102]. Moreover, the raise in life expectancy in all countries of the world increases the percentage of the population with a too high body mass index. In high-income countries this transition, the so-called “energy flipping point”, occurred already more than 50 years ago, but todays it applies to nearly every country on this planet [4].

Hunger and satiety are feelings that are coordinated by numerous hormones signaling to the brain [4]. Satiation hormones control the amount of food intake, while obesity hormones modify these signals [103]. These regulatory circles are modulated by cultural habits, stress and social influences. Taking up less calories than consumed by daily physical activity and the basal metabolic rate, *i.e.*, a negative energy balance, could solve the problem of overweight and prevent obesity. However, neuronal and hormonal control circuits have been evolutionary adapted to make hunger a prime instinct of humans and other animals. This is the main driver of feeding behavior and counteracts strongly with most attempts of reducing body weight [4]. In addition, nutrition-triggered epigenetic programming, which happens in pre- and postnatal phases, can result in an epigenetic predisposition for overweight and obesity [104].

Taken together, our nutritional habits change in the period of only two generations more drastically than in any other time in the history of our species. This time span is too short for expecting any genetic adaptations.

## 6 Genetic adaptation to dietary changes

The evolution of our species is largely driven by change in our nutrition and environment and allowed us to progress and survive. For example, we are the only species who invented, already some 780,000 years ago, the use of fire for cooking [105]. This resulted in a less microbe-burden diet that in addition was easier to digest. In this way, cooking increased the energy yield from diet and triggered the enlargement of our brain, which largely depends on glucose. In addition, we developed receptors for sweet taste [106], which allows us detecting most energy-rich food sources. Nowadays, this survival instinct unfortunately contributes to overweight and obesity [4].

Human diet is majorly composed of starch from different types and forms of grains, potatoes or other root vegetables. Starch is a plant polysaccharide that can be digested to glucose by the enzyme amylase (AMY). The *AMY* gene family is composed by *AMY1*, which is expressed and secreted by saliva producing cells, as well as by *AMY2A* and *AMY2B* that are expressed in the pancreas [107]. The *AMY1* gene copy number increased in populations, such as in Japan, where starch rich diets like rice are favored, while other populations, such as with Siberian Yakut, which primarily eat fish and meat, the copy number stayed low [108]. Today's humans have in average some 16 copies of the *AMY1* gene [107], which *via* higher AMY protein secretion into the saliva improves the digestion of starchy foods and increases the sweet sensation during eating. The *AMY1* gene amplification is a master example of positive evolutionary selection in response to an dietary trigger. Interestingly, after the domestication of the wolve some 30–40,000 years ago, the *AMY1* gene copy number in dogs also significantly increased, in order to better digest remainders from human food [109].

The alcohol dehydrogenase (*ADH*) gene cluster is another example positive evolutionary selection in humans. With the invention of agriculture the production and consumption of fermented alcoholic beverages became popular. When humans started to consume alcohol at larger quantities, there was evolutionary pressure for more genes encoding for ethanol metabolizing enzymes. Interestingly, today's populations differ significantly in their sensitivity to alcoholic drinks. However, there is evidence that the adaptation of humans to alcohol consumption already started far earlier than in neolithic times [110]. In addition, alcohol can be oxidized also in microsome by the enzyme CYP2E1 (cytochrome P450 family 2 subfamily E member 1). The actions of both ADHs and CYP2E1 affect the redox status of cells *via* the NAD/NADH ratio and the generation of ROS, *i.e.*, the evolutionary adaptation to use with alcohol an additional energy-rich food source came with the disadvantage of additional oxidative stress [111].

The introduction of agriculture as well as the migration to new geographic environments positively selected for the genes *ADAMTS19* (ADAM metallopeptidase with thrombospondin type 1 motif 19), *ADAMTS20*, *APEH* (N-acylaminoacyl-peptide hydrolase), *UBR1* (ubiquitin protein ligase E3 component n-recognin 1) and *PLAU* (plasminogen activator, urokinase) that encode for enzymes of protein metabolism [112]. Furthermore, there are examples of nutrition-related gene variants that are specific for one or several populations, such as *MAN2A1* (mannosidase α, class 2A, member 1) in East Asia and West Africa, *NCOA1* (nuclear receptor coactivator 1) in West Africa, *SLC25A20* in East Asia, *SI* (sucrase-isomaltase) in East Asia), *LEPR* (leptin receptor) in East Asia as well as the fatty acid handling *SLC27A4* and *PPARD* (peroxisome proliferator-activated receptor delta) in Europe [112]. Thus, for the best use of local resources human populations genetically adapted to their traditional diet.

The master example of a diet-driven genetic adaptation causes lactase persistence, *i.e.*, the ability to digest milk sugar (lactose) throughout the life and not only as a young breast-fed child [6,11,113]. Lactase persistence is very common in Europe, while it is basically absent in South-East Asia. Lactose is the main carbohydrate energy source for infant mammals. The intestinal enzyme LCT digests the disaccharide into galactose and glucose. The default condition in older children and adults is a significantly reduced expression of the *LCT* gene after weaning, in order to avoid competition with newborns for breast milk. When these lactase non-persistent individuals consume lactose, they often get intestinal symptoms, such as flatulence, bloating, cramps, diarrhea and nausea, as a consequence of which they avoid drinking milk.

**Fig. 3:**
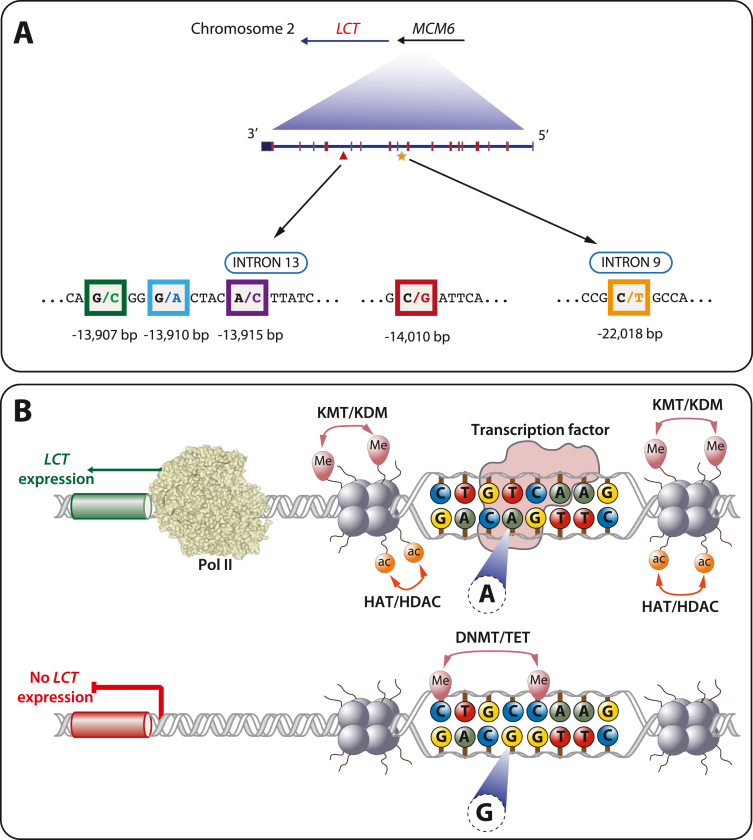
**Molecular basis of lactase persistence.** The genomic region of the genes *LCT* and *MCM6* is shown (**A**). SNPs located approximately 14 and 22 kb upstream of the TSS of the *LCT* gene, which are located within introns 13 and 9 of the *MCM6* gene, respectively, are associated with lactase persistence. The function of regulatory SNPs is schematically depicted (**B**). The SNP is part of a transcription factor binding site and provides in one allele (**top**) high affinity and in the other allele (**bottom**) low affinity for the transcription factors. In case of rs4988235 at position −13,910 relative to the *LCT* gene this is POU2F1 (POU class 2 homeobox 1). Moreover, epigenetic effects, such as histone acetylation and methylation as well as DNA methylation can affect the expression of the *LCT* gene and mediate lactase persistence.

Lactase persistence is based on several regulatory SNVs within introns 9 and 13 of the gene *MCM6* (minichromosome maintenance complex component 6), which are part of transcription factor binding sites within enhancer regions 22 and 14 kilo bp (kb) upstream of the TSS of the *LCT* gene (Fig. 3). Milk is a perfect source of carbohydrates, fat and calcium. When in the past after weaning the input from protective antibodies from breast milk was missing, the mortality of children due to infectious diseases was very high [114]. In contrast, when these children were lactase persistent, they could use milk from domesticated animals as reliable dietary source and had a significant survival advantage [115]. Therefore, regulatory SNVs leading to lactase persistence were under strong positive evolutionary selection. Archeogenomic data indicated that the variant occurred first some 5000 years ago and rapidly rose in frequency in the European population [113,116].

In summary, in addition to prominent examples like the genes *LCT*, *AMY* and *ADH* there are variants of some 100 genomic regions that monitor positive selection [112], in response to diet-driven evolutionary pressure.

## 7 Evolution of human immunity

Together with nutrient deprivation, pathogen infection is the most challenging events for human survival. During the neolithic revolution humans significantly changed their habitat by favoring vector insects, such as mosquitoes, and living together with domesticated animals, such as pigs and chicken, which are reservoirs of pathogenic microbes like bacteria, viruses and parasites. Thus, when comparing with hunters and gatherers societies the burden of infectious diseases of farmers drastically increased. These pathogens represented a strong challenge for the immune system and served as selective pressures in human evolution of the past thousands of years [117]. For example, the pandemics of the “black death”, which were caused by the bacterium *Yersinia pestis*, killed in the 14th century some 50% of the European population and led a substantial selection in the variations of immune-related genes of the survivors [118].

In order to assure the survival of our species, a sufficiently large number of individuals have to reach their reproductive and child-caring age. Therefore, evolution shaped our immune system in a way that it responds efficiently to acute infections in young people [119]. For example, variants of genes encoding for membrane immune receptors in innate and adaptive immunity were positively selected [120]. This affects not only the fight against microbes but also the control of wound healing, tissue repair, the elimination of dead and cancer cells as well as the formation of a healthy gut microbiome. There is no mechanism of evolutionary pressure beyond the age of reproduction. Therefore, it is likely that genetic traits, which had been selected to ensure fitness in early-life, may lead at older age to immunophenotypes with a high rate of chronic inflammation.

The infection with the intracellular bacterium *Mycobacterium tuberculosis* is a good example for a host-pathogen co-evolution. The first cases of tuberculosis occurred in humans some 70,000 years ago and the disease spread around the world through human migration. Nowadays, only less than 10% of infected persons develop an active form of tuberculosis. This indicates that *Mycobacterium tuberculosis* has adapted well to its host and in most cases does not harm the individual very much. Nevertheless, every year there more than one million people dying from tuberculosis. However, most of these victims are immunocompromised, *e.g.*, by old age, HIV-1 infection or other impairments.

Some 100 years ago a typical treatment of tuberculosis was the exposure with UV-B, which increases the endogenous production of vitamin D_3_. This is one of multiple examples for the interface between metabolism and immunity, which is often mediated by monocyte-derived macrophages. Another example are metabolic tissues like adipose tissue that attract macrophages and show a combined inflammatory and metabolic response. This is important, since after pathogen invasions the immune system requires lots of energy for rapid cell growth and new protein synthesis. Therefore, inflammatory mediators are able to control energy metabolism, in order to defend most efficiently against pathogens, *e.g.*, through a rapid shift from glucose oxidation to lipid oxidation. Similarly, insulin resistance can be triggered by lipids, so that glucose is preserved for the brain and erythrocytes, which depend on glucose as an energy source.

Taken together, evolutionary adaptation to nutrition and lifestyle changes, as it occurred in neolithic societies, involve immune responses and are mediated *via* the mutual control of metabolism and inflammation.

## 8 Conclusion

*Homo sapiens* lived for more than 200,000 years as hunter and gatherer in Africa and had adapted his biochemistry to this type of diet. Migrations to significantly different geographic regions within the past 75,000 years and in particular the shift to a life as farmers some 10,000 years ago created a number of evolutionary pressures. These challenges were not only a change in diet but also the burden of infectious diseases, to both of which our (epi)genome responded by adaptations. However, during the past 50 years changes in our environment and lifestyle were faster than ever in our history. This implies that many of us are not (epi)genetically prepared to the challenges of Western diet paired with the preferential sedentary lifestyle and that most of us will get sooner or later in life the diagnosis of having the metabolic syndrome. Nevertheless, we have the chance to remind our evolutionary history written down in the individual version of our (epi)genome and adapt our lifestyle accordingly.

## Financial support and sponsorship

This publication is part of a project that has received funding from the European Union's Horizon2020 research and innovation program under grant agreement no. 952601.

## Data Availability

No data was used for the research described in the article.
